# Bis[μ-1,4-bis­(4,5-dihydro-1*H*-imidazol-2-yl)benzene-κ^2^ *N* ^3^:*N* ^3′^]silver(I) dinitrate dihydrate

**DOI:** 10.1107/S1600536808001633

**Published:** 2008-01-30

**Authors:** Hua Sun, Chun-Xia Ren, Bin Shen, Zhi-Qiang Liu, Yu-Qiang Ding

**Affiliations:** a School of Chemical and Material Engineering, Jiangnan University, 1800 Lihu Road, Wuxi, Jiangsu Province 214122, People’s Republic of China

## Abstract

The reaction of 1,4-bis­(4,5-dihydro-1*H*-imidazol-2-yl)benzene (bib) with silver(I) nitrate in a 1:1 molar ratio generates the metallacyclic title complex, [Ag_2_(C_12_H_14_N_4_)_2_](NO_3_)_2_·2H_2_O, in which the bib ligand displays a *cis* configuration. Each bib ligand acts as a bidentate bridging ligand connecting a pair of Ag^I^ ions to form a [2 + 2] metallamacrocycle in which the Ag⋯Ag distance is 6.77 (2) Å. Each Ag^I^ ion has weak contacts (2.91 Å) with the nitrate anion. The uncoordinated water mol­ecules make hydrogen bonds with nitrate O atoms, forming chains. The H atoms attached to the uncoordinated nitro­gen inter­act with these chains through N—H⋯O hydrogen bonds, forming layers parallel to the (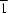
11) plane.

## Related literature

For related literature, see: Moulton & Zaworotko (2001 ▶); Nardelli (1999 ▶); Ren, Ye, He *et al.* (2004 ▶); Ren, Ye, Zhu *et al.* (2004 ▶); Ren *et al.* (2007 ▶); Toh *et al.* (2005 ▶); Zhang *et al.* (2005 ▶).
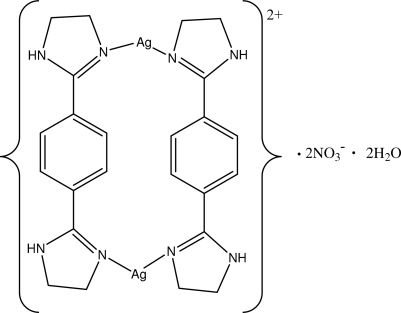


## Experimental

### 

#### Crystal data

[Ag_2_(C_12_H_14_N_4_)_2_](NO_3_)_2_·2H_2_O
*M* *_r_* = 804.34
Triclinic, 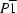

*a* = 10.3562 (19) Å
*b* = 11.053 (2) Å
*c* = 13.282 (2) Å
α = 97.496 (3)°
β = 95.354 (3)°
γ = 101.613 (3)°
*V* = 1465.3 (4) Å^3^
*Z* = 2
Mo *K*α radiation
μ = 1.40 mm^−1^
*T* = 273 (2) K
0.30 × 0.25 × 0.20 mm

#### Data collection

Bruker SMART CCD area-detector diffractometer
Absorption correction: multi-scan (*SADABS*; Bruker, 1998 ▶) *T*_min_ = 0.678, *T*_max_ = 0.767
7650 measured reflections
5316 independent reflections
3797 reflections with *I* > 2σ(*I*)
*R*_int_ = 0.017

#### Refinement

*R*[*F* ^2^ > 2σ(*F* ^2^)] = 0.049
*wR*(*F* ^2^) = 0.143
*S* = 0.92
5316 reflections
397 parameters
3 restraints
H-atom parameters constrained
Δρ_max_ = 2.17 e Å^−3^
Δρ_min_ = −0.82 e Å^−3^

### 

Data collection: *SMART* (Bruker, 1998 ▶); cell refinement: *SAINT* (Bruker, 1998 ▶); data reduction: *SAINT*; program(s) used to solve structure: *SHELXS97* (Sheldrick, 2008 ▶); program(s) used to refine structure: *SHELXL97* (Sheldrick, 2008 ▶); molecular graphics: *ORTEPIII* (Burnett & Johnson, 1996 ▶), *ORTEP-3 for Windows* (Farrugia, 1997 ▶) and *PLATON* (Spek, 2003 ▶); software used to prepare material for publication: *SHELXL97*.

## Supplementary Material

Crystal structure: contains datablocks global, I. DOI: 10.1107/S1600536808001633/dn2312sup1.cif

Structure factors: contains datablocks I. DOI: 10.1107/S1600536808001633/dn2312Isup2.hkl

Additional supplementary materials:  crystallographic information; 3D view; checkCIF report

## Figures and Tables

**Table 1:** Hydrogen-bond geometry (Å, °)

*D*—H⋯*A*	*D*—H	H⋯*A*	*D*⋯*A*	*D*—H⋯*A*
N1—H1⋯O6	0.86	2.12	2.980 (6)	173
N1—H1⋯O5	0.86	2.46	3.029 (6)	124
N3—H3⋯O1	0.86	2.13	2.915 (7)	151
N3—H3⋯O2	0.86	2.64	3.143 (6)	119
N6—H6⋯O1*W*	0.86	2.13	2.912 (6)	150
N8—H8⋯O4^i^	0.86	2.33	3.073 (7)	145
O1*W*—H11*W*⋯O2^ii^	0.85	2.12	2.852 (6)	144
O1*W*—H12*W*⋯O5^i^	0.85	2.04	2.888 (7)	178
O2*W*—H21*W*⋯O1	0.85	2.22	3.021 (7)	157
O2*W*—H22*W*⋯O6	0.85	2.21	2.973 (7)	150

Symmetry codes: (i) 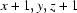; (ii) 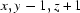.

**Fig. 1.**
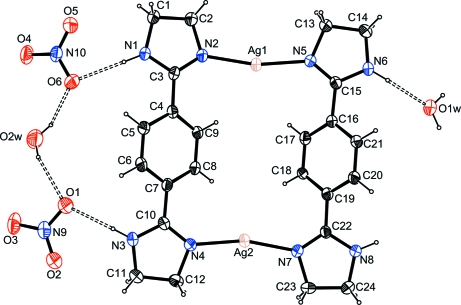
View of compound (I) with the atom-labelling scheme. Displacement ellipsoids are drawn at the 30% probability level. Hydrogen bonds are shown as dashed lines. H atoms are represented as small spheres of arbitrary radii.

**Fig. 2.**
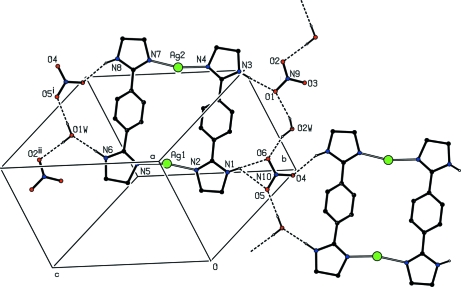
Partial packing view of [Ag2(bib)2](NO3)2.2H2O showing the hydrogen bond interactions as dashed lines. H atoms not involved in hydrogen bondings have been omitted for clarity.[Symmetry codes: (i) 1 + x, y, 1 + z; (ii) x, y - 1, 1 + z]

## supplementary crystallographic information

### Comment

Controlled assembly and crystallization of supramolecular isomers and polymorphs are an interesting challenges in the field of supramolecular chemistry and crystal engineering (Moulton *et al.*, 2001). One of the simplest example of such supramolecular isomerism may be a discrete molecule forming a one-dimensional polymer assembled in a 1/1 metal-ligand stoichiometry(Toh *et al.*, 2005; Zhang *et al.*, 2005). In our previous work, we have designed and synthesized a number of such metal complexes, including silver(I) complexes with a V-shaped ligand 1,3-bis(4,5-dihydro-1*H*-imidazol-2-yl)benzene (Ren, Ye, He *et al.*, 2004; Ren, Ye, Zhu *et al.*, 2004; Ren *et al.*, 2007) which has four potential coordinated sites with differently binding abilities. To gain more insight into the structural variation of this type of silver(I) complexes, we became interested in a new imidazole-like ligand 1,4-bis(4,5-dihydro-1*H*-imidazol-2-yl)benzene (bib). Here, we present the syntheses and structural characterizations of a new [2:2] metallocyclic silver(I) complexes, namely [Ag_2_(bib)_2_](NO_3_)_2_.2H_2_O.

The crystal structure of the title complex consists of dimeric [Ag_2_(bib)_2_]^2+^ cations, as well as NO_3_^-^ counter anion and lattice water in the solid state. As shown in Fig. 1, each pair of Ag^I^ ions in the title complex are coordinated by two nitrogen atoms from two different bib ligands resulting in a [2:2] 18-membered metallocycle with a Ag(1)···Ag(2) distance of 6.77 Å. The two bib ligands, acting in a *cis*, *cis* mode, are organized in a head-to-tail fashion and joined together by two silver ions through coordination bonds to give the metallocycles. All the Ag—N bond distances are from 2.078 (4) to 2.104 (4) Å, and agree with values found in the literature(Ren *et al.*, 2004*a*, 2004*b*, 2007). The bond angles around the Ag^I^ ion are 164.4 (2) /% and 166.5 (2) /%.

The lattice water molecules form hydrogen bonds with nitrate oxygen atoms yielding chains. The H atoms attached to the uncoordinated nitrogen interact through N—H···O hydrogen bonds with these chains forming layers parallel to the (-1 1 1) plane. (Table 1, Fig. 2).

### Experimental

All the reagents and solvents employed were commercially available and used as received without further purification.

Synthesis of Ligand bib. 1,4-Benzenedicarboxylic acid (2.31 g, 13.9 mmol), ethylenediamine (3.70 ml, 50 mmol), ethylenediamine dihydrochloride (6.64 g, 50 mmol) and toluene-*p*-sulfonic acid (0.208 g, 1.09 mmol) were added to the solvent of ethylene glycol (20 ml), and the mixture solution was refluxed for 3 h. About half of the ehylene glycol solvent was then slowly removed by distillation. The residue was dissolved in a mixture of water (40 ml) and concentrated HCl (11*M*, 3 ml). The addition of 50% aqueous NaOH gave a yellow precipitate that was purified by recrystallization. The ligand bib was obtained in 89% based on 1,4-benzenedicarboxylic acid (*ca* 2.68 g). Anal. calc. for C_12_H_14_N_4_: C, 67.27; H, 6.59; N, 26.15%. Found: C, 67.13; H, 6.87; N, 26.04%.

Synthesis of [Ag_2_(bib)_2_](NO_3_)_2_.2H_2_O. To a solution of AgNO_3_ (0.169 g, 1 mmol) in MeCN-H_2_O (*v*/*v* 1:1), an aqueous solution (2 ml) of bib (0.214 g, 1 mmol) was added. The pale-yellow solution was allowed to stand at room temperature in air avoiding illumination for a few days by slow evaporation. Colourless prismatic crystals of the title complex were obtained, which were collected by filtration washed with aqueous MeCN and dried in a vacuum desiccator over silica gel (*ca* 0.108 g, 27% yield based on AgNO_3_). Anal. calc. for C_24_H_32_Ag_2_N_10_O_8_. Main IR bands (KBr, cm_-1_): 3340*m*, 2968w, 2887w, 1615*m*, 1567*m*, 1510*m*, 1473*m*, 1365 s, 1279 s, 1184*m*, 1049w, 983w, 693w, 576w, 528w.

### Refinement

All H atoms attached to C atoms and N atom were fixed geometrically and treated as riding with C—H = 0.93 Å (aromatic) or 0.97 Å (methylene) and N—H = 0.86 Å with *U*_iso_(H) = 1.2*U*_eq_(C or N). The positions of H atoms for water molecule were calculated (Nardelli, 1999) and included in the subsequent refinement as riding with *U*_iso_(H) = 1.5*U*_eq_(O).

In the final difference map, the highest peak is 1.35 Å from Ag1 and the deepest hole is 1.30 Å from Ag2.

### Crystal data

**Table:**

[Ag_2_(C_12_H_14_N_4_)_2_](NO_3_)_2_·2H_2_O	*Z* = 2
*M**_r_* = 804.34	*F*_000_ = 808
Triclinic, *P*1	*D*_x_ = 1.823 Mg m^−^^3^
Hall symbol: -P 1	Mo *K*α radiation λ = 0.71073 Å
*a* = 10.3562 (19) Å	Cell parameters from 2137 reflections
*b* = 11.053 (2) Å	θ = 2.5–23.9º
*c* = 13.282 (2) Å	µ = 1.40 mm^−^^1^
α = 97.496 (3)º	*T* = 273 (2) K
β = 95.354 (3)º	Block, colourless
γ = 101.613 (3)º	0.30 × 0.25 × 0.20 mm
*V* = 1465.3 (4) Å^3^	

### Data collection

**Table:**

Bruker SMART CCD area-detector diffractometer	5316 independent reflections
Radiation source: fine-focus sealed tube	3797 reflections with *I* > 2σ(*I*)
Monochromator: graphite	*R*_int_ = 0.017
*T* = 273(2) K	θ_max_ = 25.5º
φ and ω scans	θ_min_ = 1.9º
Absorption correction: multi-scan(SADABS; Bruker, 1998)	*h* = −12→12
*T*_min_ = 0.678, *T*_max_ = 0.767	*k* = −11→13
7650 measured reflections	*l* = −16→15

### Refinement

**Table:**

Refinement on *F*^2^	Secondary atom site location: difference Fourier map
Least-squares matrix: full	Hydrogen site location: inferred from neighbouring sites
*R*[*F*^2^ > 2σ(*F*^2^)] = 0.049	H-atom parameters constrained
*wR*(*F*^2^) = 0.144	*w* = 1/[σ^2^(*F*_o_^2^) + (0.1055*P*)^2^] where *P* = (*F*_o_^2^ + 2*F*_c_^2^)/3
*S* = 0.92	(Δ/σ)_max_ = 0.001
5316 reflections	Δρ_max_ = 2.17 e Å^−^^3^
397 parameters	Δρ_min_ = −0.82 e Å^−^^3^
3 restraints	Extinction correction: none
Primary atom site location: structure-invariant direct methods	

### Special details

**Table:**

Geometry. All e.s.d.'s (except the e.s.d. in the dihedral angle between two l.s. planes) are estimated using the full covariance matrix. The cell e.s.d.'s are taken into account individually in the estimation of e.s.d.'s in distances, angles and torsion angles; correlations between e.s.d.'s in cell parameters are only used when they are defined by crystal symmetry. An approximate (isotropic) treatment of cell e.s.d.'s is used for estimating e.s.d.'s involving l.s. planes.
Refinement. Refinement of *F*^2^ against ALL reflections. The weighted *R*-factor *wR* and goodness of fit *S* are based on *F*^2^, conventional *R*-factors *R* are based on *F*, with *F* set to zero for negative *F*^2^. The threshold expression of *F*^2^ > σ(*F*^2^) is used only for calculating *R*-factors(gt) *etc*. and is not relevant to the choice of reflections for refinement. *R*-factors based on *F*^2^ are statistically about twice as large as those based on *F*, and *R*- factors based on ALL data will be even larger.

### Fractional atomic coordinates and isotropic or equivalent isotropic displacement parameters (Å^2^)

**Table:**

	*x*	*y*	*z*	*U*_iso_*/*U*_eq_	
Ag1	0.72496 (4)	0.29100 (4)	0.19588 (3)	0.04584 (17)	
Ag2	1.24475 (4)	0.78014 (4)	0.21805 (3)	0.04782 (17)	
N1	0.5471 (5)	0.3689 (5)	−0.0866 (4)	0.0556 (13)	
H1	0.5461	0.4098	−0.1374	0.067*	
N2	0.6169 (4)	0.3282 (4)	0.0656 (3)	0.0444 (11)	
N3	1.0950 (5)	0.8764 (5)	−0.0663 (4)	0.0562 (13)	
H3	1.0437	0.8590	−0.1234	0.067*	
N4	1.1847 (4)	0.8423 (4)	0.0829 (3)	0.0446 (11)	
N5	0.7991 (4)	0.2146 (4)	0.3188 (3)	0.0425 (10)	
N6	0.9176 (5)	0.1613 (4)	0.4484 (3)	0.0487 (12)	
H6	0.9871	0.1636	0.4901	0.058*	
N7	1.3532 (4)	0.7500 (4)	0.3484 (3)	0.0443 (11)	
N8	1.4423 (4)	0.6791 (4)	0.4819 (4)	0.0510 (12)	
H8	1.4496	0.6282	0.5248	0.061*	
C1	0.4473 (5)	0.2619 (6)	−0.0755 (4)	0.0510 (14)	
H1A	0.4548	0.1880	−0.1209	0.061*	
H1B	0.3586	0.2768	−0.0886	0.061*	
C2	0.4810 (6)	0.2495 (6)	0.0358 (4)	0.0542 (15)	
H2A	0.4184	0.2796	0.0775	0.065*	
H2B	0.4796	0.1632	0.0433	0.065*	
C3	0.6420 (5)	0.3957 (5)	−0.0059 (4)	0.0389 (12)	
C4	0.7598 (5)	0.4977 (5)	−0.0017 (4)	0.0373 (11)	
C5	0.7993 (5)	0.5387 (5)	−0.0918 (4)	0.0458 (13)	
H5	0.7540	0.4984	−0.1549	0.055*	
C6	0.9054 (5)	0.6390 (5)	−0.0876 (4)	0.0478 (14)	
H6A	0.9299	0.6661	−0.1479	0.057*	
C7	0.9754 (5)	0.6992 (5)	0.0054 (4)	0.0385 (12)	
C8	0.9353 (5)	0.6586 (5)	0.0946 (4)	0.0449 (13)	
H8A	0.9803	0.6992	0.1576	0.054*	
C9	0.8298 (5)	0.5590 (5)	0.0907 (4)	0.0430 (12)	
H9	0.8053	0.5327	0.1512	0.052*	
C10	1.0863 (5)	0.8076 (5)	0.0097 (4)	0.0391 (12)	
C11	1.2038 (6)	0.9842 (6)	−0.0381 (5)	0.0585 (16)	
H11A	1.1718	1.0604	−0.0223	0.070*	
H11B	1.2607	0.9934	−0.0918	0.070*	
C12	1.2758 (6)	0.9513 (5)	0.0572 (4)	0.0529 (15)	
H12A	1.3596	0.9307	0.0428	0.064*	
H12B	1.2929	1.0206	0.1130	0.064*	
C13	0.7201 (6)	0.0958 (5)	0.3396 (4)	0.0517 (14)	
H13A	0.7169	0.0290	0.2837	0.062*	
H13B	0.6301	0.1034	0.3487	0.062*	
C14	0.7918 (6)	0.0695 (5)	0.4389 (4)	0.0488 (14)	
H14A	0.7433	0.0841	0.4968	0.059*	
H14B	0.8055	−0.0153	0.4319	0.059*	
C15	0.9071 (5)	0.2413 (5)	0.3818 (4)	0.0407 (12)	
C16	1.0166 (5)	0.3512 (5)	0.3846 (4)	0.0379 (11)	
C17	0.9944 (5)	0.4653 (5)	0.3636 (4)	0.0404 (12)	
H17	0.9080	0.4733	0.3454	0.048*	
C18	1.0980 (5)	0.5674 (4)	0.3691 (4)	0.0349 (11)	
H18	1.0814	0.6428	0.3532	0.042*	
C19	1.2278 (5)	0.5569 (5)	0.3987 (4)	0.0380 (12)	
C20	1.2499 (5)	0.4421 (5)	0.4183 (4)	0.0422 (12)	
H20	1.3363	0.4335	0.4360	0.051*	
C21	1.1465 (5)	0.3411 (5)	0.4119 (4)	0.0413 (12)	
H21	1.1633	0.2651	0.4260	0.050*	
C22	1.3392 (5)	0.6637 (5)	0.4076 (4)	0.0359 (11)	
C23	1.4810 (6)	0.8388 (6)	0.3862 (5)	0.0539 (15)	
H23A	1.4667	0.9226	0.4044	0.065*	
H23B	1.5407	0.8402	0.3340	0.065*	
C24	1.5388 (5)	0.7930 (5)	0.4794 (4)	0.0530 (14)	
H24A	1.6260	0.7763	0.4711	0.064*	
H24B	1.5450	0.8528	0.5409	0.064*	
N9	0.9177 (6)	0.9780 (5)	−0.2608 (4)	0.0576 (13)	
O1	0.8766 (5)	0.8843 (4)	−0.2188 (4)	0.0705 (13)	
O2	1.0326 (5)	1.0387 (4)	−0.2346 (4)	0.0724 (13)	
O3	0.8422 (6)	1.0092 (5)	−0.3252 (4)	0.0875 (16)	
N10	0.4137 (5)	0.4736 (5)	−0.2932 (3)	0.0514 (12)	
O4	0.3505 (5)	0.5295 (5)	−0.3486 (3)	0.0719 (13)	
O5	0.3607 (4)	0.3683 (5)	−0.2766 (3)	0.0703 (13)	
O6	0.5283 (4)	0.5234 (4)	−0.2531 (3)	0.0635 (12)	
O1W	1.1003 (4)	0.2239 (4)	0.6372 (3)	0.0721 (13)	
H11W	1.0637	0.1943	0.6860	0.108*	
H12W	1.1775	0.2658	0.6616	0.108*	
O2W	0.5808 (5)	0.7918 (5)	−0.2779 (4)	0.0920 (16)	
H21W	0.6639	0.8127	−0.2803	0.138*	
H22W	0.5669	0.7283	−0.2473	0.138*	

### Atomic displacement parameters (Å^2^)

**Table:**

	*U*^11^	*U*^22^	*U*^33^	*U*^12^	*U*^13^	*U*^23^
Ag1	0.0469 (3)	0.0491 (3)	0.0405 (3)	0.0061 (2)	−0.00367 (19)	0.01590 (19)
Ag2	0.0504 (3)	0.0478 (3)	0.0440 (3)	0.0056 (2)	−0.00285 (19)	0.0163 (2)
N1	0.051 (3)	0.065 (3)	0.045 (3)	−0.005 (2)	−0.012 (2)	0.024 (2)
N2	0.044 (3)	0.051 (3)	0.036 (2)	0.005 (2)	−0.0003 (19)	0.014 (2)
N3	0.059 (3)	0.062 (3)	0.043 (3)	0.000 (3)	−0.007 (2)	0.024 (2)
N4	0.049 (3)	0.041 (3)	0.040 (2)	0.001 (2)	0.000 (2)	0.010 (2)
N5	0.039 (2)	0.044 (3)	0.042 (2)	0.004 (2)	−0.004 (2)	0.012 (2)
N6	0.049 (3)	0.050 (3)	0.046 (3)	0.007 (2)	−0.007 (2)	0.020 (2)
N7	0.042 (3)	0.044 (3)	0.044 (2)	0.005 (2)	−0.005 (2)	0.011 (2)
N8	0.043 (3)	0.052 (3)	0.055 (3)	−0.002 (2)	−0.010 (2)	0.023 (2)
C1	0.045 (3)	0.059 (4)	0.046 (3)	0.005 (3)	−0.006 (3)	0.015 (3)
C2	0.049 (3)	0.060 (4)	0.051 (3)	0.002 (3)	−0.002 (3)	0.019 (3)
C3	0.039 (3)	0.047 (3)	0.031 (3)	0.012 (2)	−0.002 (2)	0.007 (2)
C4	0.040 (3)	0.041 (3)	0.034 (3)	0.013 (2)	0.004 (2)	0.012 (2)
C5	0.052 (3)	0.047 (3)	0.034 (3)	0.003 (3)	−0.001 (2)	0.008 (2)
C6	0.053 (3)	0.058 (4)	0.033 (3)	0.009 (3)	0.006 (2)	0.017 (3)
C7	0.039 (3)	0.042 (3)	0.036 (3)	0.012 (2)	−0.002 (2)	0.009 (2)
C8	0.045 (3)	0.053 (3)	0.034 (3)	0.006 (3)	−0.004 (2)	0.013 (2)
C9	0.044 (3)	0.053 (3)	0.032 (3)	0.003 (3)	0.002 (2)	0.016 (2)
C10	0.044 (3)	0.040 (3)	0.036 (3)	0.010 (2)	0.007 (2)	0.012 (2)
C11	0.058 (4)	0.057 (4)	0.058 (4)	−0.002 (3)	0.004 (3)	0.025 (3)
C12	0.052 (4)	0.051 (3)	0.051 (3)	−0.003 (3)	0.008 (3)	0.015 (3)
C13	0.045 (3)	0.052 (3)	0.055 (4)	−0.001 (3)	−0.002 (3)	0.022 (3)
C14	0.052 (3)	0.049 (3)	0.047 (3)	0.004 (3)	0.008 (3)	0.019 (3)
C15	0.041 (3)	0.041 (3)	0.042 (3)	0.008 (2)	0.004 (2)	0.015 (2)
C16	0.039 (3)	0.041 (3)	0.034 (3)	0.007 (2)	0.001 (2)	0.011 (2)
C17	0.040 (3)	0.046 (3)	0.039 (3)	0.016 (2)	0.004 (2)	0.012 (2)
C18	0.038 (3)	0.027 (2)	0.040 (3)	0.007 (2)	0.001 (2)	0.010 (2)
C19	0.041 (3)	0.043 (3)	0.031 (3)	0.009 (2)	0.004 (2)	0.009 (2)
C20	0.036 (3)	0.045 (3)	0.050 (3)	0.012 (2)	0.004 (2)	0.017 (2)
C21	0.040 (3)	0.037 (3)	0.048 (3)	0.009 (2)	0.000 (2)	0.014 (2)
C22	0.034 (3)	0.040 (3)	0.038 (3)	0.012 (2)	0.005 (2)	0.014 (2)
C23	0.045 (3)	0.047 (3)	0.063 (4)	−0.006 (3)	0.002 (3)	0.013 (3)
C24	0.043 (3)	0.057 (4)	0.052 (3)	−0.001 (3)	−0.007 (3)	0.013 (3)
N9	0.073 (4)	0.060 (3)	0.045 (3)	0.027 (3)	0.003 (3)	0.010 (3)
O1	0.076 (3)	0.061 (3)	0.073 (3)	0.010 (2)	−0.001 (2)	0.020 (2)
O2	0.080 (4)	0.068 (3)	0.070 (3)	0.010 (3)	0.006 (3)	0.024 (3)
O3	0.103 (4)	0.096 (4)	0.069 (3)	0.042 (3)	−0.018 (3)	0.025 (3)
N10	0.049 (3)	0.069 (4)	0.038 (3)	0.017 (3)	0.003 (2)	0.013 (2)
O4	0.069 (3)	0.090 (4)	0.062 (3)	0.029 (3)	−0.009 (2)	0.026 (3)
O5	0.063 (3)	0.078 (3)	0.066 (3)	0.004 (3)	−0.002 (2)	0.026 (3)
O6	0.047 (3)	0.077 (3)	0.062 (3)	0.005 (2)	−0.004 (2)	0.015 (2)
O1W	0.068 (3)	0.077 (3)	0.067 (3)	0.004 (2)	−0.015 (2)	0.031 (3)
O2W	0.079 (3)	0.127 (5)	0.071 (3)	0.016 (3)	0.005 (3)	0.027 (3)

### Geometric parameters (Å, °)

**Table:**

Ag1—N5	2.087 (4)	C8—H8A	0.9300
Ag1—N2	2.104 (4)	C9—H9	0.9300
Ag2—N7	2.076 (4)	C11—C12	1.530 (8)
Ag2—N4	2.089 (4)	C11—H11A	0.9700
N1—C3	1.344 (7)	C11—H11B	0.9700
N1—C1	1.439 (7)	C12—H12A	0.9700
N1—H1	0.8600	C12—H12B	0.9700
N2—C3	1.296 (7)	C13—C14	1.542 (7)
N2—C2	1.486 (7)	C13—H13A	0.9700
N3—C10	1.339 (6)	C13—H13B	0.9700
N3—C11	1.447 (7)	C14—H14A	0.9700
N3—H3	0.8600	C14—H14B	0.9700
N4—C10	1.300 (7)	C15—C16	1.480 (7)
N4—C12	1.473 (7)	C16—C17	1.385 (7)
N5—C15	1.291 (7)	C16—C21	1.390 (7)
N5—C13	1.474 (7)	C17—C18	1.381 (7)
N6—C15	1.343 (6)	C17—H17	0.9300
N6—C14	1.466 (7)	C18—C19	1.397 (7)
N6—H6	0.8600	C18—H18	0.9300
N7—C22	1.308 (7)	C19—C20	1.386 (7)
N7—C23	1.480 (7)	C19—C22	1.459 (7)
N8—C22	1.352 (7)	C20—C21	1.371 (7)
N8—C24	1.447 (7)	C20—H20	0.9300
N8—H8	0.8600	C21—H21	0.9300
C1—C2	1.517 (7)	C23—C24	1.515 (8)
C1—H1A	0.9700	C23—H23A	0.9700
C1—H1B	0.9700	C23—H23B	0.9700
C2—H2A	0.9700	C24—H24A	0.9700
C2—H2B	0.9700	C24—H24B	0.9700
C3—C4	1.475 (7)	N9—O3	1.234 (6)
C4—C9	1.382 (7)	N9—O2	1.236 (7)
C4—C5	1.402 (7)	N9—O1	1.258 (7)
C5—C6	1.386 (7)	N10—O5	1.239 (6)
C5—H5	0.9300	N10—O6	1.240 (6)
C6—C7	1.385 (7)	N10—O4	1.243 (6)
C6—H6A	0.9300	O1W—H11W	0.8499
C7—C8	1.390 (7)	O1W—H12W	0.8500
C7—C10	1.475 (7)	O2W—H21W	0.8500
C8—C9	1.378 (7)	O2W—H22W	0.8499
			
N5—Ag1—N2	166.51 (18)	N3—C11—H11B	111.5
N7—Ag2—N4	164.37 (18)	C12—C11—H11B	111.5
C3—N1—C1	110.0 (5)	H11A—C11—H11B	109.3
C3—N1—H1	125.0	N4—C12—C11	105.0 (5)
C1—N1—H1	125.0	N4—C12—H12A	110.8
C3—N2—C2	107.1 (4)	C11—C12—H12A	110.8
C3—N2—Ag1	136.2 (4)	N4—C12—H12B	110.8
C2—N2—Ag1	116.2 (3)	C11—C12—H12B	110.8
C10—N3—C11	109.9 (5)	H12A—C12—H12B	108.8
C10—N3—H3	125.1	N5—C13—C14	105.5 (4)
C11—N3—H3	125.1	N5—C13—H13A	110.6
C10—N4—C12	107.7 (4)	C14—C13—H13A	110.6
C10—N4—Ag2	136.8 (4)	N5—C13—H13B	110.6
C12—N4—Ag2	115.5 (3)	C14—C13—H13B	110.6
C15—N5—C13	107.1 (4)	H13A—C13—H13B	108.8
C15—N5—Ag1	135.4 (4)	N6—C14—C13	100.8 (4)
C13—N5—Ag1	117.3 (3)	N6—C14—H14A	111.6
C15—N6—C14	109.1 (4)	C13—C14—H14A	111.6
C15—N6—H6	125.4	N6—C14—H14B	111.6
C14—N6—H6	125.4	C13—C14—H14B	111.6
C22—N7—C23	107.4 (4)	H14A—C14—H14B	109.4
C22—N7—Ag2	134.8 (4)	N5—C15—N6	115.9 (5)
C23—N7—Ag2	117.7 (4)	N5—C15—C16	124.9 (5)
C22—N8—C24	110.8 (4)	N6—C15—C16	119.2 (5)
C22—N8—H8	124.6	C17—C16—C21	118.5 (5)
C24—N8—H8	124.6	C17—C16—C15	122.4 (5)
N1—C1—C2	101.7 (4)	C21—C16—C15	119.1 (4)
N1—C1—H1A	111.4	C18—C17—C16	121.4 (5)
C2—C1—H1A	111.4	C18—C17—H17	119.3
N1—C1—H1B	111.4	C16—C17—H17	119.3
C2—C1—H1B	111.4	C17—C18—C19	119.6 (4)
H1A—C1—H1B	109.3	C17—C18—H18	120.2
N2—C2—C1	104.6 (4)	C19—C18—H18	120.2
N2—C2—H2A	110.8	C20—C19—C18	118.9 (5)
C1—C2—H2A	110.8	C20—C19—C22	120.1 (5)
N2—C2—H2B	110.8	C18—C19—C22	121.1 (4)
C1—C2—H2B	110.8	C21—C20—C19	121.0 (5)
H2A—C2—H2B	108.9	C21—C20—H20	119.5
N2—C3—N1	114.1 (5)	C19—C20—H20	119.5
N2—C3—C4	125.2 (5)	C20—C21—C16	120.6 (5)
N1—C3—C4	120.6 (5)	C20—C21—H21	119.7
C9—C4—C5	118.3 (5)	C16—C21—H21	119.7
C9—C4—C3	121.3 (4)	N7—C22—N8	113.6 (5)
C5—C4—C3	120.3 (5)	N7—C22—C19	126.5 (5)
C6—C5—C4	120.4 (5)	N8—C22—C19	119.9 (4)
C6—C5—H5	119.8	N7—C23—C24	106.2 (4)
C4—C5—H5	119.8	N7—C23—H23A	110.5
C7—C6—C5	120.7 (5)	C24—C23—H23A	110.5
C7—C6—H6A	119.6	N7—C23—H23B	110.5
C5—C6—H6A	119.6	C24—C23—H23B	110.5
C6—C7—C8	118.6 (5)	H23A—C23—H23B	108.7
C6—C7—C10	120.5 (5)	N8—C24—C23	101.8 (4)
C8—C7—C10	120.8 (5)	N8—C24—H24A	111.4
C9—C8—C7	120.8 (5)	C23—C24—H24A	111.4
C9—C8—H8A	119.6	N8—C24—H24B	111.4
C7—C8—H8A	119.6	C23—C24—H24B	111.4
C8—C9—C4	121.1 (5)	H24A—C24—H24B	109.3
C8—C9—H9	119.5	O3—N9—O2	121.3 (6)
C4—C9—H9	119.5	O3—N9—O1	119.7 (6)
N4—C10—N3	114.4 (5)	O2—N9—O1	118.9 (5)
N4—C10—C7	124.5 (5)	O5—N10—O6	120.0 (5)
N3—C10—C7	121.1 (5)	O5—N10—O4	119.6 (5)
N3—C11—C12	101.6 (4)	O6—N10—O4	120.4 (5)
N3—C11—H11A	111.5	H11W—O1W—H12W	107.7
C12—C11—H11A	111.5	H21W—O2W—H22W	107.7

### Hydrogen-bond geometry (Å, °)

**Table:**

*D*—H···*A*	*D*—H	H···*A*	*D*···*A*	*D*—H···*A*
N1—H1···O6	0.86	2.12	2.980 (6)	173
N1—H1···O5	0.86	2.46	3.029 (6)	124
N3—H3···O1	0.86	2.13	2.915 (7)	151
N3—H3···O2	0.86	2.64	3.143 (6)	119
N6—H6···O1W	0.86	2.13	2.912 (6)	150
N8—H8···O4^i^	0.86	2.33	3.073 (7)	145
O1W—H11W···O2^ii^	0.85	2.12	2.852 (6)	144
O1W—H12W···O5^i^	0.85	2.04	2.888 (7)	178
O2W—H21W···O1	0.85	2.22	3.021 (7)	157
O2W—H22W···O6	0.85	2.21	2.973 (7)	150

Symmetry codes: (i) *x*+1, *y*, *z*+1; (ii) *x*, *y*−1, *z*+1.
